# “Scanxiety” and a sense of control: the perspective of lung cancer survivors and their caregivers on follow-up - a qualitative study

**DOI:** 10.1186/s40359-023-01151-0

**Published:** 2023-04-17

**Authors:** Katharina Seibel, Barbara Sauer, Bernd Wagner, Gerhild Becker

**Affiliations:** 1 Department of Palliative Medicine, Faculty of Medicine, University Medical Center Freiburg, University of Freiburg, Robert-Koch-Str. 3, 79106 Freiburg, Germany; 2 Department of Palliative Care, Marienhaus Hospital, An der Goldgrube 11, 55131 Mainz, Germany

**Keywords:** Lung neoplasms, cancer survivors, Caregivers, Quality of life, Follow-up care, Survivorship care plans

## Abstract

**Objectives:**

Lung cancer survivors often suffer from physical, emotional and social long-term effects of disease and treatment. Caregivers are also affected by the cancer diagnosis throughout the course of the disease and are frequently burdened by high levels of psychosocial stress. However, little is known about how follow-up care after the completed treatment phase can help to improve long-term quality of life. In the context of patient-centred cancer care, considering the survivors’ and caregivers’ perspectives is an important step toward improving care structures. We therefore explored how lung cancer survivors and their caregivers experience follow-up examinations and their possible psychosocial effects on everyday life in order to shed light on what support is helpful for improving their quality of life.

**Material and Methods:**

25 survivors after curative lung cancer treatment and 17 caregivers underwent a face-to-face semi-structured, audio-recorded interview that was analysed using qualitative content analysis.

**Results:**

Especially burdened cancer survivors and caregivers described recurring anxiety before a follow-up appointment influencing their everyday life. At the same time, follow-up care also provided reassurance of still being healthy and helped regain a sense of security and control until the following scan. Despite possible long-term consequences in everyday life, the interviewees reported that the survivors´ psychosocial needs were not explicitly assessed or discussed. Nevertheless, the interviewees indicated that conversations with the physician were important for the success of “good” follow-up care.

**Conclusion:**

Anxiety surrounding follow-up scans, also known as “scanxiety”, is a common problem. In this study, we expanded on previous findings and found a positive aspect of scans, namely regaining a sense of security and control, which can strengthen the psychological well-being of the survivors and their families. To optimize follow-up care and improve the quality of life of lung cancer survivors and caregivers, strategies to integrate psychosocial care, like the introduction of survivorship care plans or increased use of patient-reported outcomes, should be explored in the future.

**Supplementary Information:**

The online version contains supplementary material available at 10.1186/s40359-023-01151-0.

## Introduction

In recent decades, the survival prospects of cancer patients have improved significantly in industrialized societies, and the number of people living with and after cancer is steadily increasing. Mainly responsible for these rising numbers are earlier detection, a growing and aging population, and more effective cancer treatment resulting in more people living long term after the diagnosis [1, 2]. According to estimates, this trend will continue in the coming years [2, 3]. In this context, the term cancer survivor has become commonly used and applies to every cancer patient from the time of diagnosis until the end of their life [4].

Cancer survivors may face physical, functional, emotional and social problems even years after the end of their primary cancer treatment. These issues include long-term effects of treatment like chronic pain and fatigue, but also multiple psychosocial challenges such as fear of recurrence, distress or financial worries [3, 5–9]. Lung cancer survivors face particularly burdensome challenges. They often suffer from physical long-term consequences after treatment [10] and show significantly higher psychological distress compared to cancer survivors with other common tumour entities [11, 12]. Studies have illustrated that even more than five years after diagnosis and treatment, at least one quarter of curatively treated lung cancer survivors are significantly restricted in physical ability or report significant depressive symptoms [10, 13, 14]. Caregivers of lung cancer patients are also exposed to a high psychosocial burden [15] and have the highest prevalence rate of mental illness compared to those caring for patients with other tumour types [16].

Against this background, follow-up care plays an important role in the care of lung cancer survivors and caregivers and will play an even greater role in the future. While patients in the diagnosis and treatment phase usually find sufficient contacts for medical advice within a close-knit care system [17], in the subsequent remission phase, the follow-up appointment is the central and often only place to clarify questions and problems related to the cancer.

However, looking at the current guidelines and research on follow-up care of lung cancer patients after curative treatment, it appears that the main focus is to detect any possible recurrence or second cancer as early as possible, and to identify and treat post-therapeutic somatic complications [18–21]. At the same time, there is a lack of studies on the improvement of lung cancer survivors’ long-term quality of life (QoL) through follow-up [18, 19, 22], and on the assessment of psychosocial needs in the context of follow-up care and its impact on survivors’ and caregivers’ everyday life in the family system.

## Materials and methods

### *Objectives and research questions*

To address this research gap, we conducted an interview study to explore the subjective experience of follow-up care and its possible psychosocial effects on everyday life from the perspective of lung cancer survivors and their caregivers. Since various models of survivorship care have been discussed in the last 10 years, e.g., survivorship care in a follow-up clinic or models involving primary care [23, 24], we based our understanding of follow-up care on the specific situation of survivorship care in Germany in terms of a “disease-specific care model” [23]. Such follow-up can be provided by an oncologist, internist, surgeon or general practitioner. The main goals are the earliest possible diagnosis of a recurrence or second tumour and the treatment of post-therapeutic complications with largely uniform recommendations on follow-up intervals and examination content. In addition to clinical history and examination, appropriate imaging of the lung is an important obligatory component [19, 25, 26].

In our study, we pursued the following research questions:

- What meaning do lung cancer survivors and their caregivers attribute to regular follow-up? How does regular follow-up care affect their lives?
- What do cancer survivors and caregivers experience as important about follow-up care?
- What role do psychosocial needs play in follow-up care for cancer survivors and caregivers?

### *Design and setting*

We opted for a qualitative interview study as this offers the possibility of tracing subjective experiences in depth and reconstructing attributed meanings. The study was conducted in the federal state of Baden-Wuerttemberg, Germany and was based at the Department of Palliative Medicine at the University Medical Center Freiburg.

### *Population, sampling and recruitment*

The study population was to include lung cancer survivors and caregivers, who were to be interviewed about their experience of follow-up care. Caregivers were defined as family members and friends who provided unpaid support to the lung cancer survivor during and after illness. Purposeful sampling [27] was chosen to maximize the variance of participants along the following inclusion criteria:

- 20–25 survivors after curative lung cancer treatment stage I–IIIa and 20–25 caregivers,
- 18 years or older,
- voluntary and informed consent,
- physically and mentally able to conduct an interview of 1 h,
- different histologic types of lung carcinoma (non-small cell lung carcinoma (NSCLC) and small cell lung carcinoma (SCLC)),
- different time frames for attending follow-up appointments after completion of treatment,
- different follow-up care settings.

We combined purposeful sampling with recruitment via gatekeepers. Follow-up care physicians from different settings (two Comprehensive Cancer Centers, three clinics and seven primary care practices) in Baden-Wuerttemberg cooperated in the study and addressed cancer survivors and caregivers personally along with distributing flyers. If the cancer survivor and caregiver agreed to an interview, contact details were passed on to the two female researchers (BS & KS), who explained the study to the participants again in an initial telephone meeting, answered questions and arranged specific interview appointments. This procedure made it possible to recruit participants from towns and cities as well as from rural areas. After 12 months of recruitment and transcription of the data already collected, it became clear that thematic saturation and the desired maximization of variance among participants had been achieved. The outstanding agreed interviews were nevertheless conducted and recruitment stopped after 15 months.

### *Data collection*

Semi-structured interviews were conducted face-to-face between November 2014 and January 2016 by BS and KS. We used two interview guides, which had been developed by an interdisciplinary research group at the Department of Palliative Medicine, University Medical Center Freiburg. The aim of the interview guides were to elicit subjective theories and forms of everyday knowledge. The guides were developed according to a method that includes the following steps: Collect, Review, Sort, and Subsume [28]. The research group first collected all questions of interest with regard to the research topic in a brainstorming phase without censoring. This included knowledge from the literature on “scanxiety” and cancer survivorship, in this case specifically the long-term physical, psychological and social consequences, and the role of caregivers. In a second step, the collected questions were reviewed in the group, e.g., factual questions were deleted, open-ended questions that left room for relevance to the respondents were kept, or care was taken that interviewees could actually report from their lifeworld. In a third step, the remaining questions were sorted into question bundles according to content aspects, and fourthly, a narrative stimulus/open question was sought for each question bundle, and the remaining questions were subsumed as follow-up questions (the interview guides for survivors/caregivers can be found in Online Resource A, S1). The guides specified certain topics, e.g., the meaning and procedure of follow-up care or the experience of the time before and after the follow-up examination. These were incorporated into the interview in the form of pre-formulated open questions and follow-up questions. At the same time, flexible adaptation to the narration of the interviewees was possible, i.e. no fixed sequence of questions was specified and spontaneous follow-up questions were permitted.

All interviewees received written information about the study prior to the interview, signed the consent form on the day of the interview and chose to be interviewed at home. Before the interviews, the interviewers clarified their role and research interest: At the time, BS was a doctoral student at the Department of Palliative Medicine, and KS was employed there as a social scientist. The study served as a qualification in the context of both interviewers’ doctorates and both had been trained in qualitative interviewing.

Interviewees were allowed to be accompanied by a familiar person to provide them with added security during the interview; this was the case for six interviews. Two interviews were also conducted as survivor–caregiver dyads at the request of the interviewees, and the remaining 40 were conducted as individual interviews. It cannot be ruled out that the presence of an accompanying person or the survivor–caregiver dyads led to some narratives being omitted out of consideration for the caregiver/survivor. However, our observations indicated that third party accompaniment was most prevalent when survivors were uncertain, elderly or vulnerable, and it primarily increased willingness to participate in the interview.

### *Data analysis*

After the verbatim transcription of the recorded data and pseudonymization, the interviews were analysed using thematic analysis, more precisely structured-thematic content analysis according to Kuckartz [29, 30]. The goal of this analysis approach was to create a code system that captured the essential meaning aspects of the data material while allowing for the inclusion of latent statement contents [31]. BS worked primarily on the caregiver interviews during this process (2016–2018), KS on the survivor interviews (2016 and 2021).

The first step was an intensive familiarization with the data [29]. For one third of the interviews, the structured-thematic content analysis procedure was triangulated with another text-hermeneutic method, the integrative basic method [32], which follows a sequence-analytical approach. In doing so, both researchers paid attention to interaction, syntax, semantics and narrative figures in addition to the content, and subsequently worked out central motifs and case summaries. Next, codes for the structured-thematic content analysis were created. Main codes were developed deductively based on the thematic blocks in the guidelines, while subcodes were formed inductively based on the understanding of the text obtained in the first step and an initial coding pass of the first interviews.

After completion of the code system (see Online Resource A, S2), BS and KS independently coded the caregiver and survivor interviews using the software program MAXQDA. One sixth of the interviews, however, were double-coded, and controversial coding was discussed and adjusted if necessary. Subsequently, interview passages assigned to categories were summarized and the results were outlined with regard to the research question.

### *Research ethics*

The study was approved by the Ethics Committee of the University of Freiburg in September 2014 (submission no.: 395/14) and was retrospectively registered in the German Clinical Trials Registry (trial number DRKS00006799 / date of first registration: 03/10/2015).

## Results

### *Sample*

40 of the 42 interviews conducted were included in the data corpus: 25 survivor interviews and 17 caregiver interviews. Two interviews were removed because it became clear during the interview that the inclusion criteria were not met due to mental instability. Even though no classical theoretical sampling was carried out [33] with the goal of data saturation, a heterogeneous sample was achieved through purposeful sampling (further information see Table 1 and Online Resource B, S3).

**Table 1:** Description of the study sample

Description of the sample		
	Survivors	Caregivers
**Study participants**	25	17
**Sex**		
Female	13	12
Male	12	5
**Average age** [years]	67	63
	(range: 52–58)	(range: 43–79)
**Average interview duration** [minutes]	42	40
	(range: 17–73)	(range: 23–73)
**Relationship to patient (caregiver)**		
Partner/spouse		13
Child		2
Close Friend		2
**Histology of tumor (patient)**		
NSCLC	21	
SCLC	2	
Carcinoid	2	
**Initial tumor stage (patient)**		
Stage II or below	14	
≥ Stadium III	7	
**Average duration since completion of therapy** [years]	3 (range: 0–11)	
**Follow-up setting**		
Comprehensive Cancer Center	10	
Primary care practice	8	
Clinic	7	

### *Code system*

The main codes of the established code system include: the course of the past cancer and coping to date in everyday life, meaning and procedure of follow-up care appointments, experience of the time before and after the follow-up examination and the survivors’ and caregivers’ resources (a detailed overview including subcodes can be found in Online Resource A, S2).

### *Main results*

For this article, the most important results of the main codes are summarized under the following three headings:

- Ongoing impact of curatively treated lung cancer in the family system: long-term and late effects.
- Meaning of follow-up care.
- Psychosocial needs during follow-up care.

The results are described in detail below. Quotes from the interviews related to the three headings and associated subthemes can be found in Tables 2, 3 and 4.

**Table 2:** – Quotes on “Ongoing impact of curatively treated lung cancer in the family system: long-term and late effects”

	Survivors	Caregivers
**Perceived long-term and late effects (physical, mental, social)**	**Survivor 25**: “So I’d say, it’s not like it was before. I can’t (sighs) get as much done, I get tired more quickly, […] I haven’t been able to go to work these last two years. I had to retire because of it; … I’m glad I don’t have to work anymore. I can rest.“ **Survivor 15**: “I’ve already noticed that even within the family, there has also sometimes been a lack of understanding, you know? My reactions—sometimes I was still not happy with myself, or I’m not always happy. Well, that’s not over yet. I’m not over it yet … I just feel limited by this disease.“	**Caregiver 6**: “Restrictions, total restrictions, because—she is already up and running, but the cough is still a burden and also with her back, she is in constant pain … and that puts a strain on the whole household, because she can’t do anything, it’s all on me.“ **Caregiver 1**: “[the situation due to the disease, the authors] stresses you out of course …. Psychologically and, I would also say, physically. Because you … must help more than usual. And then you are just doubly challenged.“ **Interviewer**: “And what does that mean for everyday life?” … **Caregiver 7**: “She can’t make beds anymore … it’s impossible to [reach, the authors] high things… as well as [bend, the authors] down … because then she gets dizzy and then she falls … Oh, it’s just not like it was before … that it would get as extreme as it is now, I wouldn’t have thought … it’s wonderful that the cancer is gone, but so much is so very different.“
**No impact of long-term and late effects felt**	**Survivor 23**: “I’m just very happy that I’m doing so well. Yes, and that I was so lucky. … I was also sure that it would be good afterwards [the time after therapy, the authors]. Yes, that was also the case.“ **Interviewer**: “That means that the disease has few limitations for you now…?“ **Survivor 8**: “Yes well, I’m actually not sick anymore. I am actually healthy. I’m just a lung amputee (laughs).“	**Interviewer**: “And how is your husband doing now?” **Caregiver 2**: “Good. Good, nothing has been detected so far … Now that we are convinced that it is probably over for now, right, we are doing quite well.“

**Table 3:** – Quotes on “Impact of follow-up care on the lives of survivors and caregivers”

	Survivors	Caregivers
**Impact of follow-up care on everyday life**	**Survivor 1**: “Two or three weeks before [the follow-up exam] … I get insanely nervous … then it already starts that … I’m just nervous or scared. I’m just afraid; afraid, what’s going to happen again?“	**Caregiver 7**: “When an exam is coming up, it starts three weeks before, then she is sick, seriously sick, really sick … I know for sure that it’s nothing but she feels honestly sick … all of a sudden she has such severe pain, like there are metastases … until we are there and the doctor says ‘Mrs. [survivor’s name, the authors], everything is all right’. Then it is fine, then she is healthy again. All of a sudden. Then the thoughts are all gone.“ **Caregiver 13**: “She is very excited, very excited, internally restless; she doesn’t want to show it, but through … movements and through thought processes … it is clearly noticeable. There is no need to ask … she is nervous and says, ‘Hopefully it will be okay’ and … then I always react to it and say ‘But there is nothing, what should be there?‘ … But of course that doesn’t help, the agitation is there … when it’s about your own body then … you are doubly and triply scared … but just like this depressed mood is of course the positive… when the result comes, ‘Thank God, yes, it’s great again, it’s done again’.“
**Fear of and around scan**	**Survivor 2**: “In the beginning, it’s always like you’re going to a court hearing where a death sentence might be pronounced, right?“ **Survivor 14**: “So, you wait again and by the time you are ready to get the injection [with the contrast medium, the authors] and lie down, an hour and a half has passed and the hour and a half is, in my opinion, the greatest poison for patients. Because the patient is so tense inside, to the point of no return and wonders: what’s happening? You’re not being told anything.“ **Survivor 1**: “The worst are the minutes when I’m waiting for the CT, when I’m waiting until it’s my turn. Then I think, dear God in heaven, oh please please please let me be healthy, let it be gone …. it’s just exhausting. I’m just brutally scared.“	**Caregiver 14**: “The patient always has this sword of Damocles over him, where he never knows. And I think the fear is also always there at every follow-up examination. Is it going well? Will anything be found? Am I lucky?“ **Caregiver 1**: “I sit in the waiting room and wait for her to come back. Of course, the waiting is always the worst. First of all, waiting for the examination to start and then, even worse, waiting for the examination results. Most of the time, they don’t tell you the results right away. The radiologists have the images in front of them, but they can’t tell you anything because they have to talk to the oncologist or the surgeon or whatever. And then it can happen that two or three days pass before you find out what it looks like. And that’s particularly stressful, of course.“
**Sense of security and control**	**Interviewer**: “And what does follow-up care mean to you?“ **Survivor 15**: “Certainty … I couldn’t say anything else about it now. Certainty about how things will go on … I’ve been so lucky … and to put that at risk … I don’t really want to.“ **Survivor 19**: “You have the feeling of safety and also a little bit of security and of supervision; and you’re not alone. Because if you discover something early, you can still help sooner than if it takes too long, right?“ **Survivor 8**: “Yes, it is clear, it is always certainty, because it was quickly clear that I had no metastases. … and then of course you always want to know if it’s going to stay that way… and it is already good to know that nothing is wrong. Then at least you have a sense of security, right?“	**Caregiver 17**: “For me, it is a very great security.“ **Caregiver 13**: “I personally believe that this rhythm of one year is actually … a good thing, because you simply know it is one year without the enemy coming into the picture again … Then it is simply a very clear thing.“ **Caregiver 2**: “And now [after the follow-up, the authors] we can really plan again. Well, my husband [the survivor, the authors] always said beforehand, ‘Wait and see’ in regard to vacation planning or anything else.“
**No impact on everyday life**	**Interviewer**: “Would you say that follow-up care … has an influence on your everyday life… ?“ **Survivor 23**: “Not at all. To me it’s just normal, like going to the ear doctor or going to the dentist … for me it was natural and it was logical and reasonable and clear why you do it. And it was never a big deal for me that I had to go there. It wasn’t like that. … No, not a scary thing.“	**Interviewer**: “What does it mean to you when your husband has such a follow-up examination?“ **Caregiver 8**: “Actually not very much. Well, he says, ‘I have to go there again’. But I always think, ‘Well, I’m sure it’s all right’. So I don’t drive myself crazy about it.“ **Interviewer**: “And how were you then during the time you were waiting [at the follow-up, the authors]?“ **Caregiver 15**: “Good, actually. … Because I actually think she [the survivor, the authors] is going to make it. I really believe that she’s going to make it, that there’s no cancer coming back.“

**Table 4:** – Quotes on “Communication with the physicians in the context of follow-up care” and “Psychosocial needs during follow-up care”

Communication with the physicians in the context of follow-up care		
	**Survivors**	**Caregivers**
**Positive aspects of communication with physicians**	**Survivor 13**: “I had the feeling that this is a doctor who knows a lot. I put a lot of trust in him … and over the years … it … has become a bond. A connection has grown out of it. I trusted him enormously and that strengthened me.“ **Survivor 6**: “He’s really good. He responds to you, is human, has understanding. So yes, you realize that you are in good hands. And that’s really important.“ **Survivor 12**: “He really encourages me. … and you can tell he’s happy with me, too, because I’ve come so far, right? “	**Interviewer**: “What do you find so good about it [the conversation with the doctor, the authors]?“ **Caregiver 11**: “That you are seen as a human being. Not only … the illness … And just the feeling that the doctor has a moment for me now. That is always … very important to me. Whether it concerns me or also the other person.“ **Caregiver 7**: “Well, when I have a question, I always get it answered, always. But I also don’t rest until I know what I want to know.“ **Interviewer**: “And how do you feel involved in your wife’s follow-up care as a caregiver?“ **Caregiver 1**: “As long as I can be present at the consultations if I want to, and the doctors also talk to me. Or I can also talk to the doctors and ask questions, which has been quite good so far, so I feel quite well included.“
**Negative aspects of communication with physicians**	**Survivor 17**: “I’ve had so many changes now in the last year and a half. Really, almost every time a different doctor. That’s not so pleasant.“ **Survivor 22**: “Somehow it was about smoking. I was just a heavy smoker and then he kind of barked at me (disguised voice): ‘Look at you, you look twenty years older.‘ So, the whole tone, the way he … has. That’s not my thing at all. I don’t think patients in a situation like this should be insulted like that. I thought that was harsh.“ **Survivor 13**: “And in the beginning, he never looked at me. He only ever talked to my husband, not me. And then I said, ‘I’m actually the patient, you can look at me, can’t you?‘”	**Caregiver 12**: “I would like to ask more questions sometimes. But I just have the feeling … that it goes too far.“
Psychosocial needs during follow-up care		
	**Survivors**	**Caregivers**
**No expectations of addressing psychosocial issues**	**Interviewer**: “And do you feel that you have received holistic care? […]” **Survivor 18**: “Oh boy. I mean, they just want to look at my lungs, otherwise I’m fine. Otherwise I don’t have anything… I actually don’t want to be cared for anymore. I (laughing) want to be left in peace and be healthy.“	**Interviewer**: “And do you have the feeling that you can also talk to doctors about the topic of quality of life? **Caregiver 14**: “I haven’t thought about that yet (laughs).“ **Interviewer**: “So, is that also an issue in follow-up, quality of life? Or questions you have about that?“ **Caregiver 13**: “Actually no, no … that’s never been an issue …. Your own common sense tells you that it can’t be the same as it used to be … I think many doctors simply ignore it. … The doctor would have to have a lot of time to get to grips with that.“
**Wish for psychosocial support**	**Survivor 15**: “Well, it really got me down sometimes, and every now and then I would have liked to have a contact person. A psychologist or something. But you don’t get one. I tried once to get an appointment. You can’t get one. Waiting time: half a year, three quarters of a year. Yeah, that doesn’t help me either if I’m in a bad way at the moment.“	**Caregiver 7**: “What I would recommend to anyone in such cases is a psychologist … but she [cancer survivor, the authors] herself refused …. I would recommend it to anyone … because today she really struggles. It doesn’t matter what it is, she immediately thinks that she has cancer, regardless of what she has.“ **Caregiver 13**: “And then I … found a [psychologist, the authors] and the first thing he said to me was, ‘But you know that costs a lot of money’. And I thought that was the most stupid expression possible for a psychologist … So I didn’t take him, but I didn’t find a second one and we needed one … very very much.“

### *Ongoing impact of curatively treated lung cancer in the family system: long-term and late effects*

An important theme that emerged in the analysis was the lingering impact of past cancer on the family system, which at times strongly influenced the experience of follow-up care. About half of the former patients reported long-term or late consequences in their daily lives, especially in the form of fear of recurrence and/or physical burdens. They also faced social challenges: changed daily routine due to physical limitations or redistributed/new roles in the family, social or professional environment.

The caregivers focused even more clearly on redefined and adapted roles and relationship structures in everyday family life due to the long-term consequences. This often led to a challenging dual role of being both a supporter and a fellow sufferer, and some even felt they had reached their own limits.

However, the experience of ongoing burdens did not apply to all interviewed cancer survivors. Some of the former patients and caregivers stated they had ‘come to terms’ with the past disease. These survivors reported that the disease hardly or no longer affected them in everyday life.

### *Meaning of follow-up care*

The experience of follow-up care and the importance attributed to it were often related to the ongoing long-term consequences or, respectively, to whether survivors were no longer actively affected by their illness.

#### *3.2.1 Impact of follow-up care on the lives of cancer survivors and their caregivers: anxiety and a sense of security*

More than half of the cancer survivors and a large proportion of the caregivers described tension, mental stress and anxiety—for days or even weeks before the regular follow-up appointments. This was especially true for those interviewees who were still experiencing physical and psychosocial effects of the past lung cancer in the family’s everyday life. Cancer survivors, in particular, referred to fear of recurrence, re-experiencing the disease, concerns about the future, reminders of their own mortality or fear of death, insomnia and diffuse bodily sensations associated with suspicions about tumour recurrence.

At the follow-up appointment, the waiting between imaging and receiving the result was perceived as particularly stressful. When the findings could not be discussed between the provider and the survivor on the same day, this agonizing uncertainty was prolonged until the following appointment or until a letter with the results arrived in the mail.

In addition to anxiety and tension, a second theme emerged with regard to the importance of follow-up care, namely the sense of security. Follow-up care was considered important because it provided reassurance that the cancer survivor remained cancer-free. Until the following appointment, this allowed for a sense of security that enabled survivors and caregivers to plan for the near future. The caregivers in particular stressed that “you can’t feel lung cancer”, emphasizing that medical check-ups during follow-up care were an important tool for them for providing a sense of security. The fact that follow-up care allowed for recurrence to be detected early, thereby increasing the chance of successful treatment, was also viewed positively. For some cancer survivors, attending regular appointments was also a way to actively look after their own health. After receiving positive news, everyday life could continue without the anxiety related to the scan and results.

In contrast, others in the sample who were no longer burdened by the disease did not experience this fear followed by relief. Rather, this section of the sample perceived follow-up care solely as a standard check-up procedure, in which pleasant meetings with the medical team conveyed a sense of security. In a few cases, there was even a feeling of indifference toward follow-up care.

#### *Communication with the physicians in the context of follow-up care*

In addition to the perceived meaning of follow-up care, interviewees described communication with the physicians as especially significant. Against a backdrop of emotional tension leading up to appointments, the lung cancer survivors and caregivers valued personal communication with physicians, in addition to their professional competence. Friendliness or even compassion, openness and the possibility of asking questions, encouragement and recognition on the part of the physician were all viewed as important factors. In contrast, experiencing a lack of empathy, frequently changing doctors or the unexpected departure of a follow-up physician was perceived negatively by survivors.

Among the caregivers who were present at the follow-up, the discussion with the doctor was considered an important opportunity to actively participate and access information. The conversation helped them to better understand the former patient’s condition. In addition, caregivers gained more confidence in dealing with unclear or unsettling information and benefited when allowed to voice their own concerns.

### *Psychosocial needs during follow-up care*

Despite the long-term consequences for everyday life described above, the interviews showed that physicians generally focused on the somatic aspects of the past illness during follow-up care. Questions about physical symptoms or persistent side effects of treatment were common. In addition, the interviewees reported no explicit assessment or discussion of the cancer survivors’ psychosocial situation.

A striking phenomenon, however, was that cancer survivors also did not expect physicians to address issues related to QoL and psychosocial needs. This was either because they no longer perceived limitations in their QoL or because, like the physicians, they considered somatic aspects to be the main focus of follow-up care. Furthermore, survivors cited time restrictions in the health care system. The long-term consequences were often accepted as part of the disease, and some were receiving psychological care elsewhere for other reasons. Psychosocial well-being was also viewed by some as primarily a self-care task. At times, psychosocial interventions were even negatively labelled with stigmas such as “being useless” (Survivor 19) or “dragging down” (Survivor 21). Nevertheless, some cancer survivors and caregivers did indicate that they had experienced the need for psychosocial support, either in the past or in their current situation. However, this need was not addressed in follow-up care, and it proved difficult to find providers when left to search independently.

## Discussion

### *Meaning of follow-up care for lung cancer survivors and their caregivers*

The aim of the present study was to investigate the subjective experience of follow-up and its possible psychosocial impact on everyday life from the perspective of lung cancer survivors and their caregivers. The results show: About half of our sample no longer feels impacted, or only marginally, by the past disease, and is satisfied with the follow-up care and the sense of security it provides. Accordingly, survivors and caregivers describe that follow-up care has little influence on everyday life. The remaining survivors and caregivers, on the other hand, report ongoing physical and psychosocial long-term consequences and—in addition to the experience of safety and control—also great stress in the period before the follow-up appointment.

These findings are consistent with recent survivorship research showing the successful transition from active treatment to the ‘new sense of normal’ for some cancer survivors [5, 6]. However, a relevant proportion of cancer survivors experience long-lasting psychological and/or physical symptoms, and limitations in QoL [5, 6, 23], and caregivers of cancer survivors in general continue to suffer from burdens and unmet needs [3, 8, 9]. The results of our study also indicate possible late and long-term effects in everyday life for curatively treated lung cancer survivors and their caregivers.

The experience of distress before and during follow-up appointments is another important issue typically faced by these interviewees. The term “scanxiety”, the fear of or relating to the scan, has been used increasingly in recent years. This neologism capturing the phenomenon of anxiety or stress related to cancer scans first occurred in print and social media [34–36]. Meanwhile, it has been acknowledged and discussed as a “common and important clinical problem” (35, p. 17) and has been increasingly explored in studies addressing different types of cancer and stages of cancer care [e.g., 34, 35, 37–41]. While other studies tend to emphasize the stress and anxiety associated with follow-up care appointments [34, 37, 39], or indicate that “scanxiety” is a normal and inevitable experience for advanced cancer patients [38], the present results expand on previous findings. Anxiety around the scan has an aggravating impact on the everyday lives of certain lung cancer survivors and caregivers. Nevertheless, it is important to recognize that it also provides a sense of security and control. Survivors and caregivers view follow-up care as a way to regain a sense of control and return to ‘normal’ life until the following scan.

The little available research on feasible and effective interventions for dealing with “scanxiety” primarily refers to informative-educational interventions by psychologists or nurses [e.g., 42, 43], or meditation or relaxation exercises [e.g., 44, 45] immediately prior to the scan. Our findings suggest that conversations with physicians in particular would be a way to evaluate “scanxiety” and its impact on follow-up appointments over time. Within these conversations, triggers for distress that do not require much effort or resources to eliminate, such as waiting several days for examination results, can be addressed. Moreover, the assessment of “scanxiety” could then provide indications for further psychosocial care as part of integrated care.

### *Psychosocial needs during follow-up care*

International research has shown that there is a lack of options to support cancer survivors regarding their psychosocial needs [46]. Similarly, our results show that other domains of health besides somatic aspects, such as (1) psychological well-being (e.g., depression or anxiety, fear of recurrence or the overall perception of QoL), (2) social well-being (e.g., family distress, employment or isolation), or (3) spiritual well-being (e.g., the meaning of illness, hope or uncertainty) [3, 24, 47, 48], were generally not the focus of follow-up—indicating that structured assessment and support for psychosocial needs are not commonly integrated into the follow-up care of lung cancer survivors in Germany. Based on our results, it appears that this lies with both parties, meaning that physicians do not address psychosocial needs and survivors and caregivers do not expect them to do so. For the survivors and caregivers, in addition to the reasons they give, this may be due to the fact that they have not experienced the opportunities and benefits of psychosocial support.

When one considers that up to 40% of cancer survivors have unmet psychosocial needs [46] and caregivers often experience considerable psychosocial burden [49], as also shown in this study and in the calls of guidelines and recommendations to integrate psychosocial care into comprehensive follow-up care [18, 19, 50], it is clear that more needs to be undertaken to identify the support needs of survivors and caregivers to detect those at risk [49]. One way to address these challenges and optimize follow-up care is to introduce structured survivorship care plans, which has been increasingly discussed in recent years [3, 51]. Such plans explicitly address psychosocial care by including diagnosis and treatment summaries as well as individual treatment and follow-up care planning for medical, rehabilitative and psychosocial support services [50]. Moreover, they aim to involve survivors and caregivers in a participatory manner [47] to sustainably increase their competence for managing possible long-term and late effects, which can help them to maintain their QoL as well as their physical and emotional health (e.g., [52]).

One central aspect of such care plans is the increased use of patient-reported outcomes (PROs) in follow-up care, as also recommended by the American College of Chest Physicians (ACCP) guidelines [18]). This involves continuously monitoring disease- and treatment-related limitations as well as QoL from the survivors’ and caregivers’ perspectives using validated questionnaires [53]. The identification of their needs enables the use of interventions to tackle e.g., pain, fear of progression, or social and occupational adjustment difficulties [53].

The use of PROs could also benefit physicians. Questionnaires could be filled out during waiting times or in an app at home, saving the physicians time that they often do not have in day-to-day practice for carrying out holistic anamnesis. Computer-assisted systems are particularly convenient for identifying specific health and social problems [53].

A low-threshold screening alternative to PROs relating to psychosocial issues is the use of clinical screening questions to establish a clinical impression [54]. Examples include “How burdened have you felt in the past week?“ or “How much have you felt affected by nervousness, anxiety, or tension in the past two weeks?“ ([55], p. 48).

In an ideal world, patients and caregivers are proactively involved by discussing the results of these questionnaires or screening questions in a structured and holistic way. Survivors and caregivers greatly value communication with physicians, illustrating the importance of conversation for the success of good follow-up care. By offering opportunities for their questions and problems to be addressed, health literacy [56, 57] as well as self-management can be promoted [47, 51, 54]. In this context, the physicians providing follow-up care should not only give the patients but also the caregivers the opportunity to actively participate in the conversation. In a recent qualitative study, it could be shown that caregivers of lung cancer patients consider medical appointments as their most important information opportunity, but on the other hand, often feel excluded by the clinicians and have no possibility to ask questions [58]. Especially considering the high psychosocial burden of this group as well as the knowledge that medical information helps caregivers cope with their role [59], this seems to be another important point that follow-up physicians should consider in their consultations.

However, even if physicians are unable to find time to discuss questions and psychosocial needs themselves in their everyday practice, they could always arrange help, provide information about psycho-oncological support services or refer survivors and caregivers directly to a psycho-oncologist or a self-help group, for example. Taken together, these measures can better empower survivors and caregivers, and help them to successfully manage the consequences of the disease in the long term [56].

### *Strengths and limitations*

To the best of our knowledge, the present study is one of only a few qualitative studies exploring the meaning of follow-up care among lung cancer survivors and caregivers together. This seems especially important considering that there are very few studies on burden in caregivers of patients with curative treatment intentions. In our research, we found only one study from 2016 by Kim et al. that focused on caregivers of patients receiving curative lung cancer treatment [15]. In this study, 12 weeks after the patient’s treatment was completed, caregivers actually had a higher psychosocial burden than patients. This unexpected result is also confirmed by our study: caregivers can suffer from a considerable burden even years after completion of the patient’s curative lung cancer treatment, with consequences for their QoL as well.

Another strength is that the study is based on the biopsychosocial model and deliberately draws in the perspectives of the affected survivors and caregivers as important stakeholders. Furthermore, the qualitative design made it possible to openly ask about subjective attributions of meaning and therefore to elaborate on the mix of fear and security felt in the context of “scanxiety”. This has not been explicitly highlighted to date and adds to the survivorship and “scanxiety” evidence base.

Nevertheless, despite sampling in different follow-up care contexts and purposefully seeking variance maximization, this study was specific to the survivorship care situation in Germany. The experiences of follow-up care and the continuing burdens of cancer survivors and their relatives in everyday life are certainly transferable to other countries. However, the differences between health care systems, including the organization of follow-up care and the possibilities of receiving support, must be taken into account.

In addition, more empirical evidence on “scanxiety” is needed to build on the concept and explore feasible and effective interventions [35], as well as on the structured assessment of psychosocial needs during follow-up care and ways to address them [18] as a means to sustainably improve the QoL of cancer survivors and their caregivers.

## Conclusions

Due to demographic development and the increasing numbers of cancer survivors, follow-up care will play a greater role in the future. In turn, structured, efficient and equally effective approaches for different survivor groups are needed.

Although further research is needed to justify widespread implementation of survivorship care plans [50, 60, 61] and determine successful models and approaches for psychosocial care [46], the discourse around survivorship care planning provides important impulses. Even though this can represent a challenge for the already scarce resources in the health care system, providers on site should take heed of this and reconsider follow-up care in a more comprehensive way. At least a holistic monitoring approach could be included in follow-up care to determine whether specific long-term and late effects or needs are present. Burdened lung cancer survivors and their caregivers could benefit greatly from this.

Follow-up care can provide many opportunities for lung cancer survivors and caregivers to participate and be empowered, leading to a sense of security and control and the chance to regain temporary agency and the ability to plan. Open, empowering communication along with a good relationship and competent care can act as a safety net in both directions, whether by accompanying cancer survivors into the later phases and providing continued support or in the event of a recurrence and the need for renewed treatment or palliation.

## Electronic supplementary material

Below is the link to the electronic supplementary material.

Supplementary Material 1

Supplementary Material 2

## Data Availability

The datasets (transcripts) generated and analysed during the current study are not publicly available as we assured the participating interviewees that the transcripts would not be shared in their entirety, but only individual quotes would be cited in publications. The coding tree and coded sample citations can be requested from the corresponding author.
