# Supplementary material for: “Scanxiety” and a sense of control: the perspective of lung cancer survivors and their caregivers on follow-up - a qualitative study
Source: BMC Psychol. 2023 Apr 17;11:119. doi: 10.1186/s40359-023-01151-0 (PMC10111662; doi:10.1186/s40359-023-01151-0)
Supplement: Supplementary file 1 — Supplementary Material 1 [file 40359_2023_1151_MOESM1_ESM.pdf]

Online Resource A

Table 1: Interview guides with question prompts and possible follow-up questions for a) survivors and b) caregivers

a) Survivors	b) Caregivers
Question prompt 1<br>**Perhaps you can briefly introduce yourself to start and then talk about how your past cancer has progressed to date.**<br><br>Possible follow-up questions:<br>▪ Were there any particular turning points or experiences in your illness that were especially important? What were they?<br>▪ Can you describe in more detail how you are currently feeling?	Question prompt 1<br>**Perhaps you can briefly introduce yourself to start and then talk about how your loved one's past cancer has progressed to date.**<br><br>Possible follow-up questions:<br>▪ Were there any particular turning points or experiences in your relative's illness that were especially important to you? What were they?<br>▪ Can you describe in more detail how your relative is currently doing?<br>▪ How do you feel as a caregiver with regard to your loved one's illness?<br>▪ What influence does your loved one's illness have on your everyday life?
Question prompt 2<br>**Can you tell me more about it: What does follow-up mean to you?**<br><br>Possible follow-up questions:<br>▪ How do you feel about the follow-up: positive/negative? Why?<br>▪ What is the importance of the follow-up examinations with regard to the past cancer?	Question prompt 2<br>**Can you tell me more about it: What does follow-up mean to you as a caregiver?**<br>Possible follow-up questions:<br>▪ What does a follow-up examination mean for your loved one?<br>▪ How do you feel about the follow-up: positive/negative? Why?<br>▪ What is the importance of the follow-up examinations with regard to the past cancer?
Question prompt 3<br>**So tell me, what is a follow-up appointment like for you?**<br><br>Possible follow-up questions:<br>▪ What exactly happens during the examination?<br>▪ What do you experience as positive about the examinations? What do you find challenging?<br><br>▪ How do you experience the doctors in the follow-up examination?<br>▪ How do the doctors tell you the results of the examination?	Question prompt 3<br>**So tell me, what is a follow-up appointment like for you?**<br><br>Possible follow-up questions:<br>▪ Do you accompany your loved one to the follow-up examinations? (If not: Does someone else accompany your loved one?)<br>▪ What exactly happens during the examination?<br>▪ What do you experience as positive during the examination? What do you find challenging?

▪ Does someone accompany you to the examination? Who?<br>▪ To what extent have people who are important to you and support you in making decisions - e.g. your family or friends - been involved in your follow-up care so far?<br><br>▪ How do you feel about these examinations taking place regularly?<br><br>▪ Is there anything that scares you with regard to the follow-up?<br>▪ How do you deal with the fear? / What helps you here?<br><br>▪ Do you have any specific suggestions for improvement? Which ones?<br><br>▪ What influence do the follow-up examinations have on your everyday life? Your private life? Your job?<br>▪ How do you feel before the follow-up?<br>▪ How do you feel after the follow-up?<br><br>▪ And when you think about follow-up care: do you feel you receive holistic care? Why/why not?<br>▪ What does the keyword quality of life mean to you personally against the background of the past cancer?<br>▪ Do you feel that (what is important to you in terms of quality of life) has a place in follow-up care?<br>▪ To what extent have you talked about the topic of quality of life/about these aspects with your doctor within the follow-up care?<br><br>▪ Do you still have unanswered questions or topics in relation to the past cancer? What are they?	▪ How do you and your loved one experience the doctors during the follow-up examination? How do the conversations between you, your loved one and the doctors go?<br>▪ As a caregiver, how do you feel involved in the care of your loved one? Would you have wished for something different?<br><br>▪ How do you feel about these examinations taking place regularly?<br><br>▪ What scares you with regard to the examinations? How do you deal with it? / What helps you?<br><br>▪ Do you have any specific suggestions for improvement? Which ones?<br><br>▪ How would you describe that: What impact do follow-up examinations have on your daily life as a family? Do you talk about the exams as a family/couple? And on your professional life?<br>▪ How do you feel as caregiver before the follow-up? How do you feel after the follow-up examination?<br>▪ And how do you experience your loved one before the examination? How do you experience your loved one after the examination? What does your loved one tell you about the examination?<br><br>▪ What does the keyword quality of life mean to you and your loved one against the background of the past cancer?<br>▪ As a caregiver: Do you feel that (what is important to you in terms of quality of life) has a place in follow-up care?<br>▪ To what extent have you and your loved one talked about the topic of quality of life with your doctor within the follow-up care?<br><br>▪ As a caregiver, do you have any unanswered questions regarding the past cancer of your loved one that have not yet been clarified/addressed? What are they?

	▪ Do you feel like you have a safety net around you through your doctor's care?
Question prompt 4<br>**How do you handle follow-up examinations in terms of people close to you?**<br>Possible follow-up questions:<br>▪ How does your environment (work, circle of friends/acquaintances) deal with the examinations?<br>▪ Do you talk about the upcoming examinations/and the results?<br>▪ How do you experience the reactions?<br>▪ What is helpful? / What do you perceive as a potential burden?	Question prompt 4<br>**How do you handle follow-up examinations in terms of people close to you? And your loved one?**<br>Possible follow-up questions:<br>▪ How does your environment (work, circle of friends/acquaintances) deal with the examinations?<br>▪ Do you talk about the upcoming examinations/and the results?<br>▪ How do you experience the reactions?<br>▪ What is helpful? / What do you perceive as a potential burden?
Question prompt 5<br>**If you now think about the period since the treatment: What has been particularly beneficial for you since then?**<br>Possible follow-up questions:<br>▪ What gives you strength?<br>▪ Did you possibly also learn through the follow-up to pay attention to yourself and what is good for you? Can you provide an example?<br>▪ What do you wish for your future?	Question prompt 5<br>**If you now think about the period since the treatment of your loved one: What has been particularly beneficial for you since then?**<br>Possible follow-up questions:<br>▪ What gives you strength?<br>▪ Did you possibly also learn through the follow-up to pay attention to yourself and what is good for you? Can you provide an example?<br>▪ What do you wish for your future?

Table 2: Code system of a) the survivor interviews and b) caregiver interviews

a) Code system “Survivors“	b) Code system “Caregivers“
1. Course of past cancer and treatment of survivor	1. Course of the survivor's disease
2. Current coping with the past cancer illness & well-being/long-term effects of the survivor in everyday life	2. Experiencing illness and stress as a caregiver in everyday life
	3. Role of the caregiver in everyday life and in the previous medical history
3. Resources and sources of strengths of the survivor	4. Resources and sources of strengths of the caregiver
	5. Coping strategies of the caregiver
	6. Quality of life in the family system
4. Meaning of follow-up care for the survivor	7. Meaning of follow-up care for the caregiver
5. Procedure of the follow-up examinations<br>a. Undergoing the procedure<br>b. Waiting<br>c. Discussion of the examination result<br>d. Experience/importance of doctors and medical staff<br>e. Quality of life/psychosocial care<br>f. Involvement of caregivers<br>g. Satisfaction with follow-up care/suggestions for improvement	8. Procedure of the follow-up examinations
	9. Role of the caregiver in regard to follow-up care
6. Experience of the time before and after the follow-up examination	10. Experience of the time before and after the follow-up examination in the family system
7. Dealing with the issue of follow-up examinations with regard to family/friends/acquaintances	11. Dealing with the issue of follow-up examinations with regard to family/friends/acquaintances

“Scanxiety” and a sense of control: The perspective of lung cancer survivors and their caregivers on follow-up - a qualitative study.

BMC Psychology. Katharina Seibel (1), Barbara Sauer (1), Bernd Wagner (2), Gerhild Becker (1)

(1) Department of Palliative Medicine, University Medical Center Freiburg, Faculty of Medicine, University of Freiburg, Robert-Koch-Str. 3, D-79106 Freiburg, Germany
(2) Department of Palliative Care, Marienhaus Hospital, An der Goldgrube 11, D-55131 Mainz, Germany

Corresponding author: katharina.seibel@uniklinik-freiburg.de
