# Supplementary material for: “Scanxiety” and a sense of control: the perspective of lung cancer survivors and their caregivers on follow-up - a qualitative study
Source: BMC Psychol. 2023 Apr 17;11:119. doi: 10.1186/s40359-023-01151-0 (PMC10111662; doi:10.1186/s40359-023-01151-0)
Supplement: Supplementary file 2 — Supplementary Material 2 [file 40359_2023_1151_MOESM2_ESM.pdf]

Online Resource B

Table 1: Sample characteristics

	Survivors	Caregivers	Sample (n=42)
**Sex**			
Female	13	12	25
Male	12	5	17
**Age**			
<50	0	2	2
50-60	4	3	7
60-70	12	6	18
70-80	8	6	14
>80	1	0	1
**Follow-up care setting**			
CCC	10	7	17
Clinic	7	3	10
Medical practice	8	7	15
**Years of follow-up since therapy**			
<1	4	2	6
1	6	3	9
2	5	4	9
3	4	3	7
4	1	1	2
5 und >5	5	4	9
**Histology of the survivors' tumors**			
NSCLC	20	-	-
SCLC	2	-	-
Carcinoid	2	-	-
Unknown	1[1]	-	-
**Initial tumor stage** [2]			
< Stadium I	1	-	-
Stadium I	9	-	-
Stadium II	4 (2 without carcinoids)	-	-
Stadium IIIA	6	-	-
≥ Stadium IIIB	2[3]	-	-
Unknown	3[4]	-	-

"Scanxiety" and a sense of control: The perspective of lung cancer survivors and their caregivers on follow-up - a qualitative study. BMC Psychology. Katharina Seibel (1), Barbara Sauer (1), Bernd Wagner (2), Gerhild Becker (1)
(1) Department of Palliative Medicine, University Medical Center Freiburg, Faculty of Medicine, University of Freiburg, Robert-Koch-Str. 3, D-79106 Freiburg, Germany
(2) Department of Palliative Care, Marienhaus Hospital, An der Goldgrube 11, D-55131 Mainz, Germany
Corresponding author: katharina.seibel@uniklinik-freiburg.de

[1] The patient was treated curatively in an initial stage. However, a pathological examination of the tumor tissue after the operation was not conducted - at the patient's request.
[2] The staging was done according to the 8th edition of the TNM classification and staging of the UICC (Union internationale contre le cancer), cf. Leitlinienprogramm Onkologie (Deutsche Krebsgesellschaft, Deutsche Krebshilfe, AWMF, http://leitlinienprogramm-onkologie.de/Lungenkarzinom.98.0.html) 2018, p. 108-112.
[3] Two patients with a tumor stage > IIIA were deliberately allowed in the sample as an exception.
[4] In one patient, after curative treatment of an initial tumour stage, no staging was carried out – at the patient's request. In the case of the other patients, no clear staging could be made on the basis of the clinical information. However, the recruiting physicians confirmed the curative treatment of the patients in each case when asked by telephone.
